# Sentinel surveillance for human enterovirus 71 in Sarawak, Malaysia: lessons from the first 7 years

**DOI:** 10.1186/1471-2458-6-180

**Published:** 2006-07-07

**Authors:** Yuwana Podin, Edna LM Gias, Flora Ong, Yee-Wei Leong, Siew-Fung Yee, Mohd Apandi Yusof, David Perera, Bibiana Teo, Thian-Yew Wee, Sik-Chi Yao, Sik-King Yao, Andrew Kiyu, Mohd Taha Arif, Mary Jane Cardosa

**Affiliations:** 1 Institute of Health & Community Medicine, Universiti Malaysia Sarawak, Jalan Datuk Musa, Kota Samarahan, Sarawak, Malaysia; 2 Sarawak Health Department, Jalan Tun Abang Haji Openg, Kuching, Sarawak, Malaysia; 3 B Teo's Specialist Clinic, Kuching, Sarawak, Malaysia; 4 TY Wee Specialist Clinic for Children, Kuching, Sarawak, Malaysia; 5 Faculty of Medicine and Health Sciences, Universiti Malaysia Sarawak, Jalan Datuk Musa, Kota Samarahan, Sarawak, Malaysia

## Abstract

**Background:**

A major outbreak of human enterovirus 71-associated hand, foot and mouth disease in Sarawak in 1997 marked the beginning of a series of outbreaks in the Asia Pacific region. Some of these outbreaks had unusually high numbers of fatalities and this generated much fear and anxiety in the region.

**Methods:**

We established a sentinel surveillance programme for hand, foot and mouth disease in Sarawak, Malaysia, in March 1998, and the observations of the first 7 years are described here. Virus isolation, serotyping and genotyping were performed on throat, rectal, vesicle and other swabs.

**Results:**

During this period Sarawak had two outbreaks of human enterovirus 71, in 2000 and 2003. The predominant strains circulating in the outbreaks of 1997, 2000 and 2003 were all from genogroup B, but the strains isolated during each outbreak were genetically distinct from each other. Human enterovirus 71 outbreaks occurred in a cyclical pattern every three years and Coxsackievirus A16 co-circulated with human enterovirus 71. Although vesicles were most likely to yield an isolate, this sample was not generally available from most cases and obtaining throat swabs was thus found to be the most efficient way to obtain virological information.

**Conclusion:**

Knowledge of the epidemiology of human enterovirus 71 transmission will allow public health personnel to predict when outbreaks might occur and to plan interventions in an effective manner in order to reduce the burden of disease.

## Background

Hand, foot and mouth disease (HFMD) is a common acute viral illness that primarily affects infants and young children, and often occurs in clusters or outbreaks. It is characterized by rapid onset of fever and sore throat, accompanied by vesicles and ulcers on the gums, tongue, buccal mucosa and palate. Punctate and usually transient skin lesions appear on the palms, soles and occasionally on the buttocks, knees or other areas. While the fever and rash may subside rapidly, the mouth lesions may last more than a week, and virus may continue to be shed for several weeks [1]. In temperate countries HFMD occurs during the summer but in the tropics HFMD can occur at any time during the year.

The major causative agents of HFMD are coxsackievirus A16 (CVA16), human enterovirus 71 (HEV71) and coxsackievirus A10 (CVA10) of the genus *Enterovirus *in the family *Picornaviridae *[2]. Other enteroviruses isolated from HFMD cases are the other species A human enteroviruses such as coxsackievirus A (CVA) 4, CVA5, CVA6 and CVA7, and coxsackievirus B (CVB) 1, CVB2, CVB3 and CVB5 [2-4]. Unlike other aetiological agents of HFMD that normally cause mild disease, HEV71 infection has been reported to cause neurological disease manifesting as aseptic meningitis, encephalitis or poliomyelitis-like acute flaccid paralysis[5]. First isolated from a child suffering from encephalitis in California in 1969, HEV71 was further isolated from 23 cases with severe neurological disease in California during the next three years[3]. Historically, HEV71-associated outbreaks have been reported in Australia in 1972[6], Japan in 1973 and 1978[7], Bulgaria in 1975[8] and Hungary in 1978[9].

In the past decade, countries in the Asia-Pacific region have experienced an increased occurrence of HEV71-associated HFMD outbreaks[10]. HEV71 outbreaks have been reported in Sarawak in 1997, Taiwan in 1998, Perth in 1999, then in Singapore, Korea, Malaysia and Taiwan in 2000 [11-17].

In an outbreak of HEV71 in 1997 in Sarawak, a state of Malaysia on the island of Borneo, a cluster of unusual paediatric deaths due to encephalitis and cardiac failure was observed[12,13]. This raised a lot of fear and anxiety and because of the heightened concern about HEV71 in Sarawak, we implemented a sentinel surveillance programme for HFMD beginning in March 1998. This programme was set up as part of the operational functions of the Sarawak Health Department and was approved by the Director of Health. The principles of the Helsinki Declaration were followed throughout the surveillance operation.

Our aims were to investigate the epidemiology of this common childhood disease in Sarawak, and to determine if there were any differences in the patterns of transmission of HEV71, CVA16 and other aetiological agents of HFMD. It was also the aim of this programme to provide data of practical value for doctors and public health personnel with a view to efficient and effective virological surveillance of HEV71, in particular, to provide an early warning system for HEV71 outbreaks. This paper describes the preliminary observations from our surveillance programme from March 1998 through June 2005.

## Methods

### Sentinel clinic programme

In early 1998 after discussion with a number of community paediatricians, our team set up a protocol for a sentinel surveillance programme for HFMD. The doctors who had consented to actively participate were provided with a standard reporting and specimen collection form, sterile swabs and virus transport medium, a telephone number for obtaining assistance for transport of specimens to the laboratory and a facsimile number to report cases to the Health Department. All sentinel clinic doctors obtained parental consent before swabs were taken. Sentinel clinic doctors were provided with feedback on viruses isolated from their patients and contact was maintained through both outbreak and inter-outbreak periods to assure doctors that the surveillance programme was active and ongoing. Data obtained in this manner were expected to provide accurate information about disease trends and molecular epidemiology of the relevant viruses.

### Patients and specimens

Three specialist paediatric clinics located in the towns of Kuching and Sibu in the state of Sarawak actively participated in this study from March 1998. In 2000 we included a fourth specialist clinic in Sibu. Two government polyclinics in Kuching and Sibu also participated as sentinel clinics. All children presenting to the sentinel clinics with a history of oral or other skin lesions typical of HFMD were enrolled into the surveillance study, and throat and rectal swabs were obtained from each child enrolled in the first 18 months. Where possible, swabs were also obtained from mouth ulcers, vesicles and other skin lesions. After a preliminary analysis of data from the first 18 months, the protocol was modified to require only throat swabs from sentinel clinics. Rectal swabs were optional and doctors were requested to provide vesicle swabs whenever possible. Specimens were to be transported on ice to the laboratory in 2 ml of viral transport medium (VTM) where they were vortexed, freeze-thawed and aliquoted.

### Virus isolation

Since the primary objective of programme was a sentinel surveillance system for HEV71 HFMD, we inoculated specimens into human rhabdomyosarcoma (RD) cells susceptible to both CVA16 and HEV71. It was not a particular objective of this exercise to identify the minor causative agents known to be associated with HFMD and hence we did not include multiple cell lines as part of our virus isolation protocol. RD cell cultures normally showed the characteristic enterovirus CPE in 2 to 10 days and were harvested after the monolayer showed extensive CPE. A blind passage was done with all cultures showing no CPE after 10 to 14 days.

### RNA extraction

RNA was extracted from all culture harvests using Tri Reagent LS (Molecular Research Centre, Cincinnati, OH, USA) according to the manufacturer's instructions. The dry RNA pellet was dissolved in 20 μl of sterile ultra high quality RNase-free water and stored at -80°C until use.

### RT-PCR for identification of enterovirus

The presence of enterovirus RNA in culture fluids was determined by a previously described pan-EV RT-PCR method[18] with some modifications. The duration of all the steps in the PCR was reduced to one minute and the final extension was reduced to 5 minutes.

### RT-PCR for specific identification of HEV71

From 1998 through 2002, specimens positive using the pan-EV primers were tested for the presence of HEV71 genome by RT-PCR using the primers 159S and 162A, which anneal to the VP1 gene of HEV71. Dr. Mark Pallansch (Centers for Disease Control, Atlanta) generously made the primer sequences available to us prior to publication[19]. All PCR products were sequenced to confirm the identification. In 2002, we changed our protocol for identification of HEV71 due to problems of misidentification of local strains of CVA16 as HEV71 using the primer set 159S/162A[20]. Currently, HEV71 specific primers designed in-house are used for specific identification of HEV71[20]. All primers used are listed in Table 1.

### DNA sequencing of PCR products

Sequencing reactions were performed using the Big Dye Terminator Cycle Sequencing Kit version 3.0 or 3.1 (Applied Biosystems, Foster City, CA, USA).

### Serotype identification of non-HEV71 enteroviruses isolated

Molecular serotyping of non-HEV71 enteroviruses isolated was carried out using the methods and sequences published by Oberste and colleagues[21] and Chu, Ishiko and colleagues[22,23]. Prior to 2002, serotyping of selected non-HEV71 enteroviruses was performed exclusively according to Oberste's method. When Ishiko's method[23] was published in 2002, we made a comparison of the methods by serotyping new isolates using both methods. We determined that for human species A enteroviruses circulating in our region, Ishiko's primers gave identical serotype identification to that obtained using Oberste's method[21]. Since there was no discrepancy between the methods, we modified Ishiko's primers to convert the method from a semi-nested to a non-nested method for ease of use. Our modifications were verified and described by Cardosa and colleagues[24]. We then retrospectively retested all non-HEV71 enteroviruses isolated prior to 2002 using the modified method.

### Confirmation of HEV71 identification

A subset of isolates identified by sequencing of VP4 were subjected to confirmation by using VP1 specific primers and DNA sequencing of the PCR products, as described previously[24].

### Phylogenetic analysis

DNA sequences of VP1 and VP4 gene products generated by RT-PCR from isolates were used in this analysis essentially as previously described[24]. The software package ClustalX[25] was used for alignment and to generate a bootstrapped phylogenetic tree using the neighbour joining method according to Saitou and Nei[26].

### Primers and DNA sequences

All primers used in the methods described above are listed in Table 1. All new DNA sequences used in the phylogenetic analysis but hitherto unpublished have been deposited in GenBank and have the accession numbers AY794032, AY794033, AY794035 to AY794042. All other sequences used are from previous publications by our own as well as other groups[17,20,22-24,27]. Detailed protocols, sample collection methods and other practical information have been placed in the public domain through our APNET (The Asia-Pacific Enterovirus Surveillance Network) website[28].

### Statistical analysis

Statistical analysis was performed using the software package JMP Statistics version 5.01(SAS Institute Inc., USA) and Prism 4 for Macintosh (Graphpad Software, Inc., USA).

## Results

### The first 18 months

The first provisional protocol we provided to the sentinel clinics for the collection of specimens required both throat and rectal swabs and vesicle or ulcer swabs where possible. Results from virus isolation studies of specimens obtained from both sentinel clinics as well as hospitals during this period were used to review the protocol that was originally implemented. A total of 579 specimens from 263 children with a clinical diagnosis of HFMD were received during the 18-month period from March 1998 through August 1999. The age of the children ranged from 6 months to 13 years, with 153 (58.2%) males and 110 (41.8%) females.

All specimens received were subjected to virus isolation. Fifty specimens from 44 children, of a total of 579 (8.6%) specimens, were too heavily contaminated with bacteria. Twenty four of the contaminated specimens were rectal swabs, 19 were throat swabs and 7 were from various skin lesions. All remaining uncontaminated cell culture harvests were tested for enteroviruses by using the pan-EV set of primers and 235 of the 529 (44.4%) specimens tested yielded an enterovirus, but only 15 of the 235 (6.4%) enteroviruses were HEV71. These specimens were from 259 children and an enterovirus was isolated from 153 (59.1%) children. Only 6 (3.9%) of the children had HEV71.

From this early set of specimens, we were able to isolate an enterovirus from 44% of the throat swabs, 40% of the rectal swabs, 44% of the mouth ulcers and 66% of the vesicle swabs. Clearly vesicle swabs are very useful specimens, but only 18% of the children had had vesicle swabs taken because not all children presented with skin lesions filled with abundant fluid. Since throat swabs provided a reasonably high yield of enterovirus isolates, we made the decision in 2000, to require throat swabs as the primary specimen from the sentinel clinics, with vesicle swabs where possible, while rectal swabs were not required. This served to reduce the laboratory workload during an outbreak.

### Epidemiological curves – 7 years (1998–2005)

**Figure 1:**
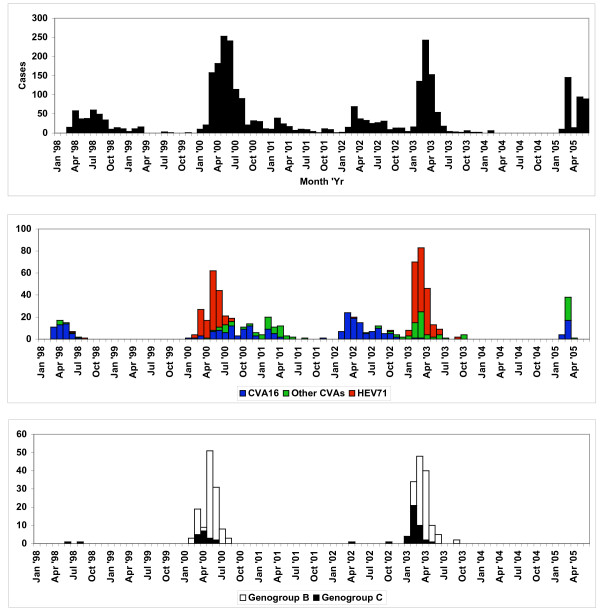
**Distribution of HFMD cases reported by Sarawak sentinel clinics and enteroviruses isolated from March 1998 through June 2005**. The top panel shows the distribution of cases of HFMD seen by our sentinel clinics. The middle panel shows the distribution of different enteroviruses isolated from specimens submitted by our sentinel clinics. The bottom panel shows the distribution of HEV71 isolates alone.

Our laboratory received 4290 specimens from 2950 children from March 1998 through June 2005, with a male to female ratio of 1.35:1. The histogram in the top panel of Figure 1 shows the distribution of HFMD cases seen in our sentinel clinics during this period. There have clearly been two large outbreaks of HFMD in 2000 and 2003 (bottom panel of Figure 1), with some sporadic activity between these peaks. The dominant enterovirus serotype isolated during both the outbreaks was HEV71 as shown in the middle panel of Figure 1. CVA16 was always isolated during HEV71 outbreaks as well but was also isolated in inter-outbreak periods. Other species A human enteroviruses such as CVA2, CVA4, CVA5, CVA10 and CVA12 were also isolated in inter-outbreak periods.

### Phylogenetic analysis of HEV71 strains isolated

**Figure 2:**
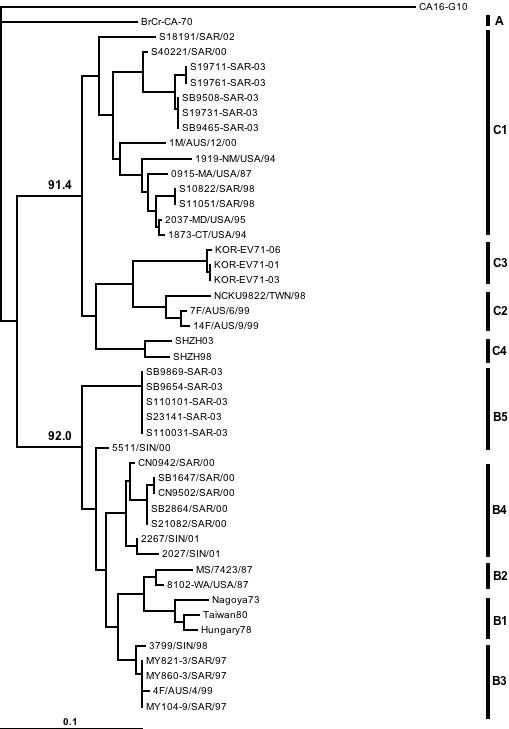
**Phylogenetic tree generated from the VP4 gene, showing relationships between HEV71 isolated in different years**. The bar denotes relative phylogenetic distance and indicates proportion of nucleotide substitutions per site. The sequence of the VP4 gene region (position 747–953, relative to HEV71 strain 7423-MS-87) is used for the generation of this tree.

**Figure 3:**
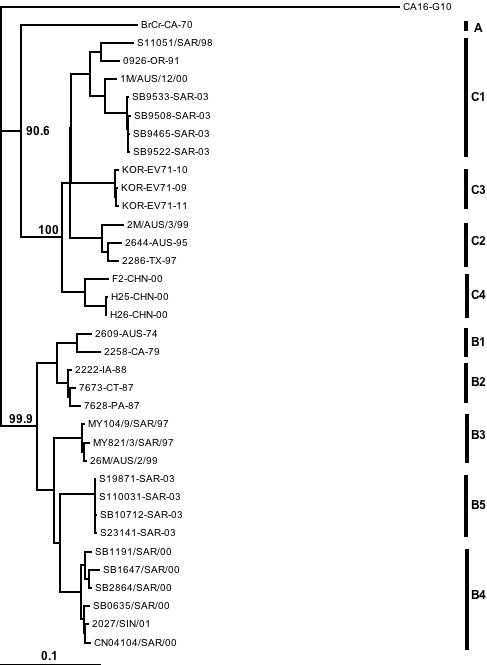
**Phylogenetic tree generated from the VP1 gene, showing relationships between HEV71 isolated in different years**. The bar denotes relative phylogenetic distance and indicates proportion of nucleotide substitutions per site. The sequence of the VP1 gene region (position 2442–3332, relative to HEV71 strain 7423-MS-87) is used for the generation of this tree.

Phylogenetic analysis of the HEV71 strains isolated in Sarawak from 1998 to 2005 show that both genogroup B and genogroup C strains circulated in Sarawak during this period (see Figures 2 and 3). We have used both VP4 and VP1 genes in the phylogenetic analysis in order to be certain that there is no major discrepancy in genotyping associated with using VP4 and VP1 gene regions. Furthermore, we wish to provide both options to other groups who may wish to compare their data with ours since it is known that many groups may still use VP4 sequencing as a first step in molecular identification of human enteroviruses. Although both genogroup B and genogroup C HEV71 strains co-circulated in Sarawak, the predominant genogroup in both the HEV71 outbreaks of 2000 and 2003 was genogroup B. Besides co-circulating with genogroup B strains during outbreaks, genogroup C viruses also appeared sporadically between outbreaks along with other species A human enteroviruses. We never isolated a genogroup B HEV71 in non-outbreak periods. The distribution of genogroup B and genogroup C HEV71 strains during the surveillance period is shown in the bottom panel of Figure 1.

The phylogenetic trees in Figures 2 and 3 also show that the genogroup B viruses circulating in Sarawak during the surveillance period were from 2 distinct clusters that were also distinct from the genogroup B virus that caused the large outbreak in Sarawak in 1997. These clusters have been named subgenogroup B3 (1997 outbreak), B4 (2000 outbreak) and B5 (2003 outbreak). The genogroup C viruses circulating in Sarawak were all from subgenogroup C1.

### Genogroup shift observed in the outbreak of 2003

By 2003 we had already established and fine-tuned our methods for rapid identification and genotyping of HEV71 and all genotyping of virus isolates was performed immediately after confirmation of HEV71. To our surprise we observed a shift in genogroup from C to B in the early phase of the outbreak. The first cases of HEV71 associated HFMD presenting to our sentinel clinics were determined to have HEV71 of genogroup C1. The number of HFMD cases being admitted to hospitals also began to rise in early February and continued to rise through the next month (data not shown). We found that the number of HEV71 isolates recovered from both patients admitted to hospitals and patients presenting to our sentinel clinics during this period followed this trend. HEV71 of genogroup B5 was isolated in small numbers in the first 6 weeks of the outbreak (epidemiological week 5 to 10) and as the numbers of genogroup C1 viruses isolated began to decline, the numbers of genogroup B5 viruses isolated began to rise, showing a dominance of genogroup B5 viruses from epidemiological week 11 onwards. To illustrate this, we have taken a snapshot of data available at epidemiological week 20 [see additional file 1] to show the virological situation at that point in time. Thus, in 2003 there were in fact 2 clusters of HEV71 cases emerging. The main outbreak was associated with HEV71 genogroup B5, but this was preceeded by a smaller cluster of cases infected with HEV71 genogroup C1.

### Rapid rise in the number of HFMD cases during an HEV71 outbreak

**Figure 4:**
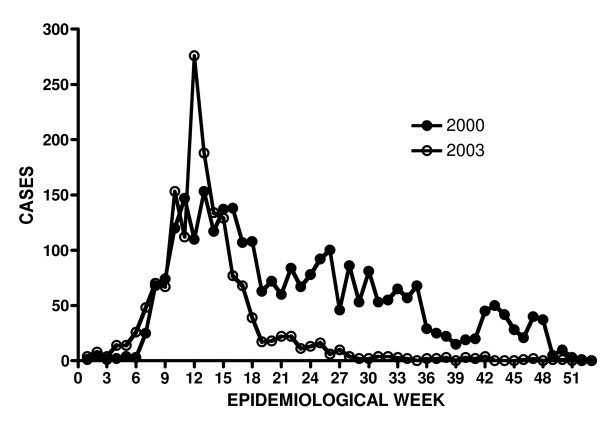
HEV71 outbreaks of 2000 and 2003 by epidemiological week.

The HFMD epidemiological curves for the outbreak years 2000 and 2003 were plotted according to epidemiological week (Figure 4) and show clearly that the first HFMD cases began to be seen early in the year. By week 7 a clear rise in the number of cases was seen. This early rise in cases differs from the summer outbreaks seen in temperate countries, and we suggest that the HEV71 outbreaks in Sarawak preceed the summer outbreaks of countries in the northern hemisphere in each year. In 2000 the HFMD outbreak stretched to the end of the year, peaking between weeks 11 to 13. The 2003 outbreak was cleaner, rising around the same time as the 2000 outbreak, but peaked at week 12 and declined sharply to very few cases by week 19. However, the epidemiological curves of HEV71 cases were similar in both 2000 and 2003. The number of HEV71 cases in 2000 dropped sharply by the end of June (by week 27) being replaced largely by CVA16 until the end of the year (see Figure 1). In 2003, the number of HEV71 cases declined sharply by the end of April (by week 18) and were no longer detected by the end of June, coinciding with the last HFMD cases seen that year. Interestingly this outbreak coincided with the SARS outbreak in the region and the public health measures put into place during this time evidently served to control the transmission of enteroviruses as well.

### Community paediatric clinics – detailed study of cases

A detailed analysis was done on data collected from two sentinel clinics, coded S1 and S2, which had sent samples to our laboratory consistently and reliably throughout the seven-year study period. A total of 2570 specimens were collected from 1894 cases during the 7 years. Of the 1894 cases, specimens from 1804 (95%) were subjected to virus isolation. A total of 2272 specimens were subjected to virus isolation, thus ensuring that the majority of specimens from the majority of cases were tested (88.4% of specimens from 95% of cases).

### a. Virus isolation

An analysis of the proportion of the different types of specimens and the virus yield obtained is shown in Table 2. More than 2000 specimens from outbreak and non-outbreak periods were tested from 1998 to 2005. Enteroviruses were grown from 21.6% of those tested. Throat swabs comprised 72.3% of the total number of specimens tested and 25.4% of these yielded enteroviruses. Detailed information about the enterovirus serotypes isolated during this surveillance programme is also provided [see additional file 2]. Although on the whole, the virus isolation success rate was much lower than anticipated from the results for the first 18 months, it remained the case that throat swabs were more useful than the rectal swabs which yielded non-polio enteroviruses in only 5.8% of the samples tested. It should be noted however, that the first 18 months of the study coincided with an inter HEV71 epidemic period, with mostly CVA16 and non-HEV71 species A human enteroviruses causing HFMD. We have compared the virus isolation yields during HEV71 outbreak (2000 and 2003) years with the yields during an HFMD outbreak caused by non-HEV71 enteroviruses (2002) and we found that we successfully isolated virus from 40% of specimens collected in 2002 but only 20% of viruses during the HEV71 outbreak years, suggesting that HEV71 is more difficult to isolate than CVA16 and other species A enteroviruses. The virus isolation rate in the first 18 months (44%) is therefore comparable to that obtained later, when HEV71 was not circulating.

### b. Age distribution

There were 491 children from whom a non-polio enterovirus was isolated. Of these, 8 were excluded from the analysis because of missing information on their age at presentation. The children ranged in age from 18 days to 155 months, with a mean of 32.2 months and a median of 27.5 months. There were 3 dominant serotypes of enteroviruses isolated from these 483 children and we asked the question if different serotypes of enteroviruses caused infection in children of different ages. Table 3 shows the mean ages of the children who had CVA16, HEV71 and CVA10 infection. Comparison of means for each pair using an unpaired t test at an alpha of 0.05, showed that there was no significant difference in the mean ages of the children in the different groups (CVA10 versus CVA16: P = 0.0872; CVA10 versus HEV71: P = 0.1800; CVA16 versus HEV71: P = 0.6992).

## Discussion

Following the 1997 outbreak of EV71 associated HFMD in Sarawak, Malaysia, the Health Department installed a sentinel surveillance programme with the expectation that we would be able to study epidemiological trends and begin to predict when to expect outbreaks with sufficient accuracy in order to implement public health interventions to reduce the burden of the disease. Although the surveillance programme is still ongoing in Sarawak, we have sought to glean some preliminary information from the data generated over the first 7 years of the programme. During this time there have been 2 outbreaks of HEV71 in Sarawak with smaller clusters of HFMD associated with CVA16 and other species A human enteroviruses occurring concomitantly with as well as independently of the two HEV71 outbreaks. A recent report on a similar surveillance programme in Yamagata Prefecture in Japan (1998–2003) suggests that in Yamagata there is frequent importation of HEV71 from surrounding countries seeding the clusters of cases seen annually in this community[10]. The HEV71 strains in this study were isolated from small clusters of cases that tended to be seen in the summer months while in our situation we observed outbreaks of HEV71 every 3 years with cases being seen much earlier in the year, well before the northern summer. In 2003 both Sarawak and Yamagata experienced a large outbreak and in both situations, a genogroup shift from C to B was noted.

It is interesting that in Sarawak, of the genogroup C viruses, only genogroup C1 strains have been observed, while genogroup B viruses appear to be changing from outbreak to outbreak, suggesting that it is likely that genogroup B viruses are evolving within Borneo and that the outbreaks we have experienced are being seeded from within rather than from imported viruses. Since the outbreaks in Sarawak typically begin early in the year, it is also possible that genogroup B strains generated in Sarawak may seed HEV71 outbreaks in the region, which typically occur later than the Sarawak outbreaks. This temporal sequence of regional outbreaks is also true of those occurring in Singapore and in Peninsula Malaysia.

The data we have obtained through 7 years of our sentinel surveillance programme for HFMD in Sarawak have provided useful clues to understanding the epidemiology of HEV71 in the state. It is clear that the appearance of HEV71 associated HFMD in sentinel clinics signals the start of an outbreak, but the rise in the number of cases is so rapid that this approach is not a suitable early warning system. In 2003 there were only 5 weeks between the time the first HEV71 cases were seen and the peak of the outbreak. Clearly this could be explained by rapid and effective response by the public health teams, but we have no way to know.

Alternatively, the 3-year cycle of HEV71 outbreaks we have observed could, if verified in the coming years, provide public health officials with the relevant information to plan and to implement their intervention programmes to reduce the disease burden in the years when an HEV71 outbreak is expected. Although this is not expected to prevent the outbreaks entirely, effective public health measures put into place early enough can limit the spread, reduce mortality and reduce the burden on the community and the health system.

It is important to note that epidemiological curves showing HFMD alone, without distinguishing the infecting agent for each case, can stretch broadly over many months, with non-HEV71 enteroviruses continuing to be isolated after cessation of HEV71 activity. This was especially evident in 2000, when HEV71 associated fatal cases were reported in neighbouring Singapore in September and October 2000[29], and the media attention surrounding these events generated a high index of suspicion in Sarawak as well. No HEV71 was isolated in Sarawak after August that year, but numerous CVA16 continued to be isolated until the end of 2000. Thus even though sociological factors affect the shape of the HFMD epidemiological curves in Sarawak, epidemiological curves specifically showing genogroup B strains of HEV71 were consistently sharp and well defined in 2000 and 2003.

The mean and median age of children with HFMD was 36 months and 30 months respectively, but the mean ages did not differ between the groups infected with the different serotypes. It is thus intriguing that HEV71 has caused much larger and sharper outbreaks than either CVA16 or CVA10. This suggests that HEV71 has the capacity to spread rapidly through the susceptible population and then become quiescent in the community. In the third year after any HEV71 outbreak, the whole cohort of children under 3 years of age has not been exposed to HEV71 and all of these children are then susceptible, providing the conditions for another sweeping transmission of HEV71 through the community. The annual birth cohort in Sarawak is 48 to 49 thousand and thus in 3 years there are up to 150,000 susceptible children in the state.

According to the trends we have reported, we expect that the next outbreak of HEV71 in Sarawak will be in 2006. At the time of writing we have already begun to pick up HEV71 cases in our sentinel programme and from past experience, an outbreak in Sarawak is often followed by outbreaks in other countries in the region. We have therefore decided to put our data into the public domain in order that other public health practitioners in the Asia Pacific region may benefit from this experience and prepare for a spread of HEV71 in the region once again in the months to come.

## Conclusion

The main conclusions arising out of this preliminary report are described below:

a. HEV71 outbreaks have occurred every 3 years in Sarawak starting in 1997. All the 3 outbreaks (1997, 2000 and 2003) have been caused by genogroup B viruses and furthermore, each of the 3 outbreaks has been associated with genogroup B viruses that are genetically distinct from each other.

b. HEV71 of subgenogroup C1 has been isolated throughout the 7 years of the surveillance programme and are closely related to each other and to genogroup C1 viruses isolated elsewhere. Sarawak has so far not experienced large HFMD outbreaks caused by HEV71 of genogroup C1. Indeed HEV71 of subgenogroup C1 behave much like other species A enteroviruses, occurring sporadically throughout the surveillance period.

c. In Sarawak, occurrence of HEV71 genogroup B infections is tightly clustered, with cases rising and falling very rapidly.

## Competing interests

The author(s) declare that they have no competing interests.

## Authors' contributions

All the virology and molecular biology was conducted by the team from Universiti Malaysia Sarawak. Members of the Sarawak Health Department provided logistic support in the collection of specimens and data from the community, and also in the communication of trends and public health measures to the primary care doctors. The surveillance system was conceived of by MTA, planned and executed by MJC, FO and AK. BT and TYW were key players in the primary healthcare setting. All authors have read and approved this manuscript.

## Pre-publication history

The pre-publication history for this paper can be accessed here:



## Supplementary Material

Additional File 1 **Genogroup shift during the 2003 outbreak**. This data shows how one genogroup of HEV71 replaced another during an HEV71 outbreak. Closed bars represent HEV71 genogroup C1 and hatched bars represent HEV71 genogroup B5. The left Y axis indicates numbers of isolates. The closed circles represent percentage of genogroup B5 viruses isolated and the percentage is indicated on the right Y axis. Click here for file

Additional File 2 **Enteroviruses isolated from different specimen types**. This table provides information about all the different enterovirus serotypes isolated from different types of specimens collected from our 2 most active sentinel clinics during the course of this surveillance programme. Click here for file

## Figures and Tables

**Table 1:** Primers used for identification of enterovirus

**Primer**	**Sequence (5'-3')**	Orientation (position)*	**Amplification of**
EVP-2 [22]	CGGAACCGACTACTTTGGGTGACCGT	Sense (538–563)	HEV71 VP4 gene.
VP2-REV [24]	TTCCAATACCACCCCTTGGATGA	Antisense (1195-1173)	HEV71 VP4 gene.
159 [19]	ACYATGAAAYTGTGCAAGG	Sense (2385–2403)	HEV71 VP1 gene.
162 [19]	CCRGTAGGKGTRCACGCRAC	Antisense (2869-2850)	HEV71 VP1 gene.
161 [19]	CTGGGACATAGAYATAACWGG	Sense (2766–2785)	HEV71 VP1 gene.
NP1A [19]	GCICCICAYTGITGICCRAA	Antisense (3355-3336)	HEV71 VP1 gene.
MAS01S [20]	ATAATAGCAYTRGCGGCAGCCCA	Sense (2355–2377)	partial VP1 gene.
MAS02A [20]	AGAGGGAGRTCTATCTCYCC	Antisense (2731-2712)	partial VP1 gene.
MD91 [18]	CCTCCGGCCCCTGAATGCGGCTAAT	Sense (450–474)	partial 5UTR.
MD90 [18]	ATTGTCACCATAAGCAGCCA	Antisense (603-584)	partial 5UTR.

*Position relative to the genome of HEV71 strain 7423-MS-87 (GenBank accession number U22522)

**Table 2:** Enterovirus yield from different specimen types over 7 years

Specimen type	Overall	Number tested (% tested)	Number positive	% positive (of tested)
Throat swab	1778	1643 (92.4)	418	25.4
Rectal swab	344	242 (70.3)	14	5.8
Vesicle swab	355	315 (88.7)	50	15.9
Oral ulcer swab	73	53 (72.6)	7	13.2
Unknown	20	19 (95.0)	2	10.5

Total	2570	2272 (88.4)	491	21.6

**Table 3:** Age of children with HFMD due to different causative agents

	Number	Mean (age in months)	Std Error	Lower 95%	Upper 95%
CVA10	79	30.0	1.69	25.6	34.4
CVA16	170	34.8	2.21	31.6	37.9
HEV71	170	33.9	1.69	30.5	37.2
