# Supplementary material for: Sentinel surveillance for human enterovirus 71 in Sarawak, Malaysia: lessons from the first 7 years
Source: BMC Public Health. 2006 Jul 7;6:180. doi: 10.1186/1471-2458-6-180 (PMC1543637; doi:10.1186/1471-2458-6-180)
Supplement: Additional File 2 — Enteroviruses isolated from different specimen types. This table provides information about all the different enterovirus serotypes isolated from different types of specimens collected from our 2 most active sentinel clinics during the course of this surveillance programme. [file 1471-2458-6-180-S2.doc]

Supplementary Table 1

Enteroviruses isolated from different specimen types

| SPECIMEN TYPE | SEROTYPE | NUMBER ISOLATED |
| --- | --- | --- |
| Rectal | CA6 | 1 |
| Rectal | CB3 | 1 |
| Rectal | CVA10 | 1 |
| Rectal | CVA16 | 7 |
| Rectal | EV71 | 4 |
| Throat | CA12 | 1 |
| Throat | CA2 | 1 |
| Throat | CA4 | 8 |
| Throat | CA5 | 8 |
| Throat | CA6 | 18 |
| Throat | CA8 | 18 |
| Throat | CVA10 | 78 |
| Throat | CVA14 | 1 |
| Throat | CVA16 | 142 |
| Throat | CVA5 | 4 |
| Throat | CVA6 | 2 |
| Throat | EV | 1 |
| Throat | EV71 | 136 |
| Ulcer | CVA16 | 4 |
| Ulcer | EV71 | 3 |
| Vesicle | CVA10 | 1 |
| Vesicle | CVA16 | 20 |
| Vesicle | E14 | 1 |
| Vesicle | EV71 | 28 |
| unknown | EV71 | 2 |
