# Supplementary material for: Sentinel surveillance for human enterovirus 71 in Sarawak, Malaysia: lessons from the first 7 years
Source: BMC Public Health. 2006 Jul 7;6:180. doi: 10.1186/1471-2458-6-180 (PMC1543637; doi:10.1186/1471-2458-6-180)
Supplement: Supplementary file 2 [file 1471-2458-6-180-S1.doc]

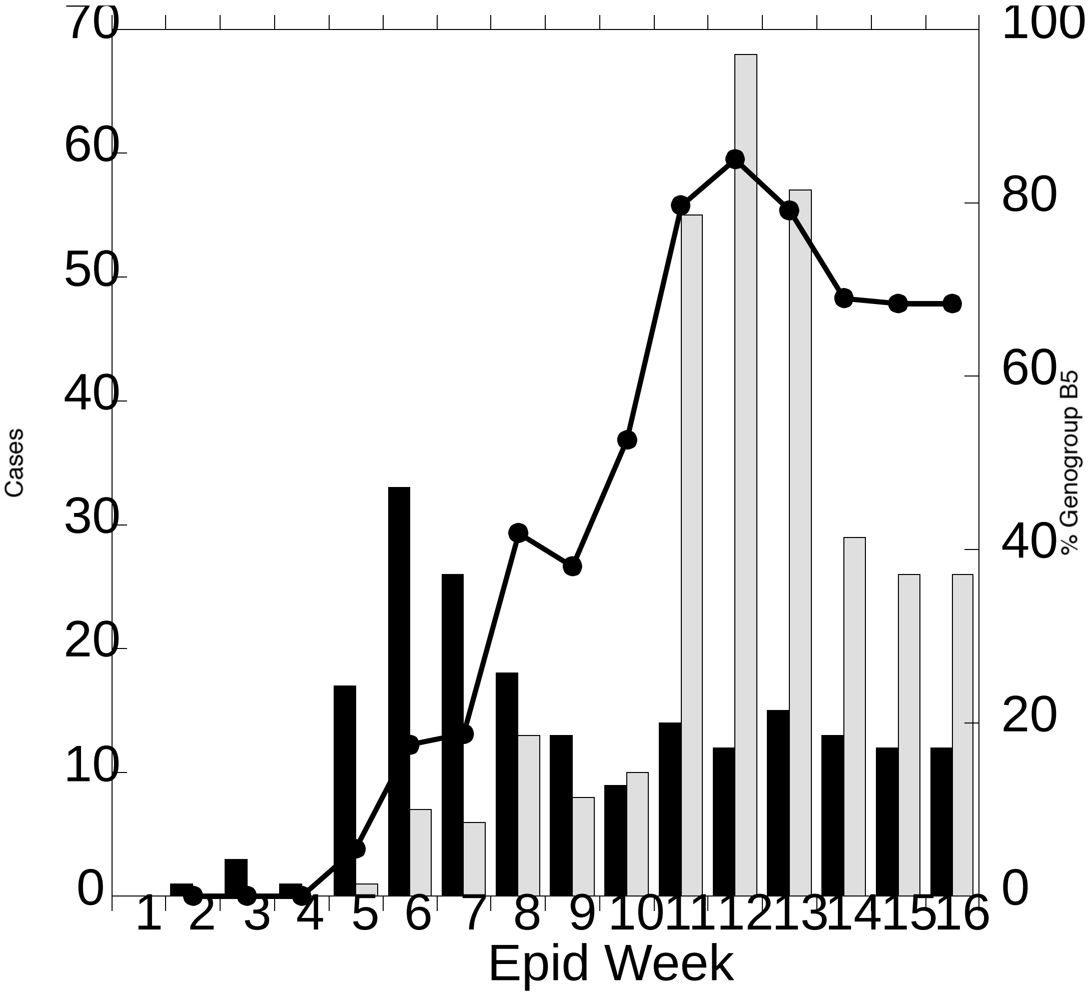

Cases
% Genogroup B5
Epid Week
0
10
20
30
40
50
60
70
0
20
40
60
80
100
1
2
3
4
5
6
7
8
9
10
11
12
13
14
15
16
